# Supplementary material for: PIWIL2 downregulation in colon cancer promotes transposon activity and pro-tumorigenic phenotypes
Source: Biol Open. 2025 Sep 4;14(9):bio061942. doi: 10.1242/bio.061942 (PMC12444863; doi:10.1242/bio.061942)
Supplement: Supplementary information [file biolopen-14-061942-s1.pdf]

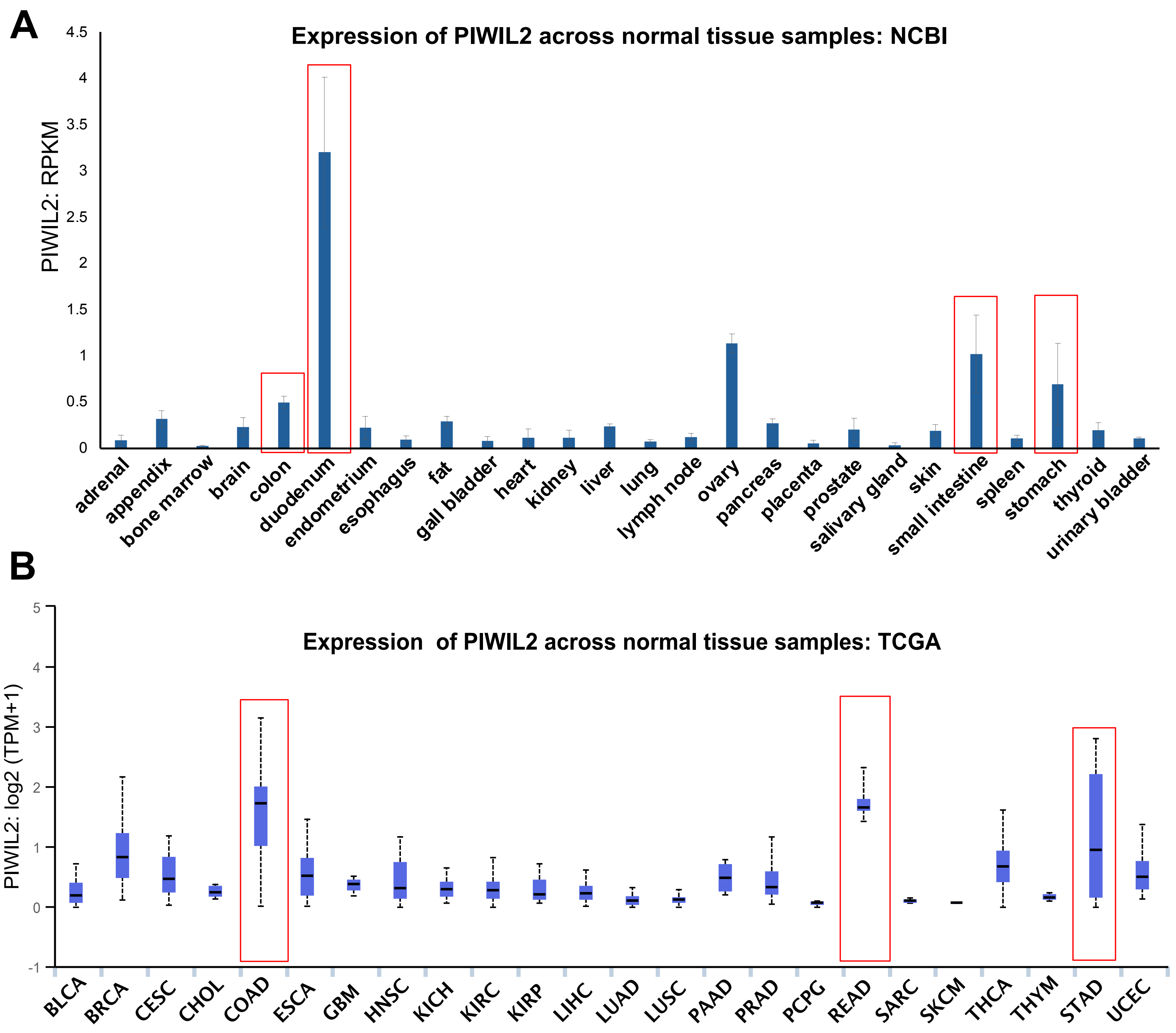

Fig. S1. PIWIL2 is highly expressed in normal somatic tissues particularly of gastrointestinal origin. A) RNA-seq data of normal tissues (project PRJEB4337) for PIWIL2, derived from NCBI and from the related PIWIL2 entry (Gene ID: 55124). Mean RPKMs (Reads Per Kilobase of exon per Million mapped reads) and error bars are shown from n=2 (duodenum, ovary, pancreas, urinary bladder), n=3 (adrenal, appendix, brain, endometrium, esophagus, fat, gall bladder, liver, salivary gland, skin, stomach), n=4 (bone marrow, heart, kidney, placenta, prostate, small intestine, spleen, thyroid), n=5 (colon, lung, lymph node) samples per indicated tissue. B) The UALCAN web-based tool was used to perform analysis of TCGA RNA-seq data to determine PIWIL2 expression across multiple normal tissue samples. Box plots show median log2TPM (transcripts per kilobase million), upper-lower quartile, and maximum-minimum distribution. Tumor types and number of samples queried: bladder urothelial carcinoma (BLCA; n=19), breast invasive carcinoma (BRCA; n=114), cervical squamous cell carcinoma and endocervical adenocarcinoma (CESC; n=3), cholangiocarcinoma (CHOL; n=9), colon adenocarcinoma (COAD; n=41), esophageal carcinoma (ESCA; n=11), glioblastoma multiforme (GBM; n=5), head and neck squamous cell carcinoma (HNSC; n=44), kidney chromophobe (KICH; n=25), kidney renal clear cell carcinoma (KIRC; n=72), kidney renal papillary cell carcinoma (KIRP; n=32), liver hepatocellular carcinoma (LIHC; n=50), lung adenocarcinoma (LUAD; n=59), lung squamous cell carcinoma (LUSC; n=52), pancreatic adenocarcinoma (PAAD; n=4), prostate adenocarcinoma (PRAD; n=52), pheochromocytoma and paraganglioma (PCPG, n=3), rectum adenocarcinoma (READ; n=10), sarcoma (SARC; n=2), skin cutaneous melanoma (SKCM; n=1), thyroid carcinoma (THCA; n=59), thymoma (THYM; n=2), stomach adenocarcinoma (STAD; n=34), and uterine corpus endometrial carcinoma (UCEC; n=35). Tissues showing the highest expression in both datasets are highlighted in red boxes and are of gastrointestinal origin.

Biology Open • Supplementary information

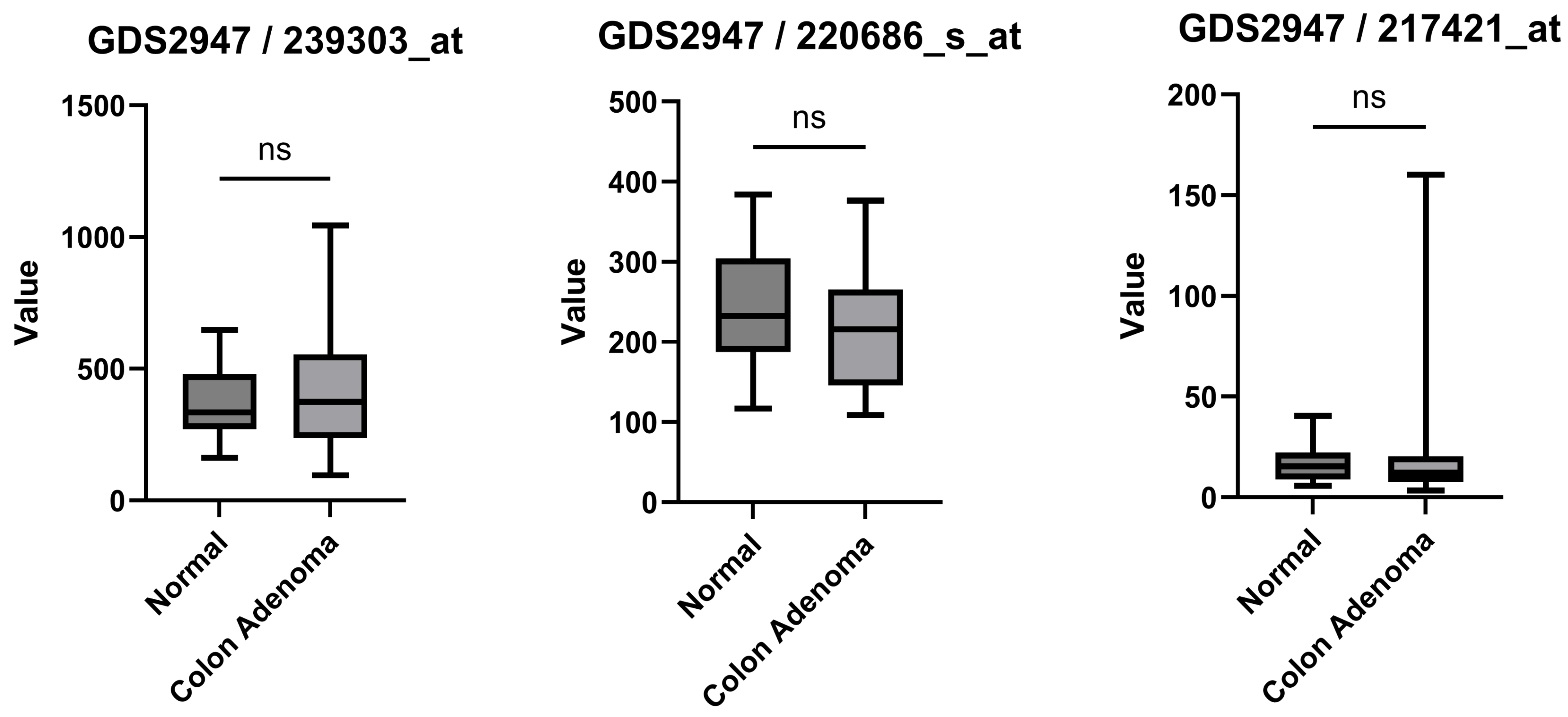

Fig. S2. PIWIL2 is not differentially expressed in colon adenomas compared to normal samples. The colon adenoma datasets used are available from the Gene Expression Omnibus (GEO): GDS2947/239303_at, GDS2947/220686_s_at and GDS2947/217421_at. N=32 samples normal and adenoma were included in all cases; a Welch's t-test was used to compare normal vs colon adenoma samples within a dataset. The box and whisker plots show the median, interquartile range, minimum and maximum values of PIWIL2 expression for each dataset, displayed in arbitrary units between datasets.

Biology Open • Supplementary information

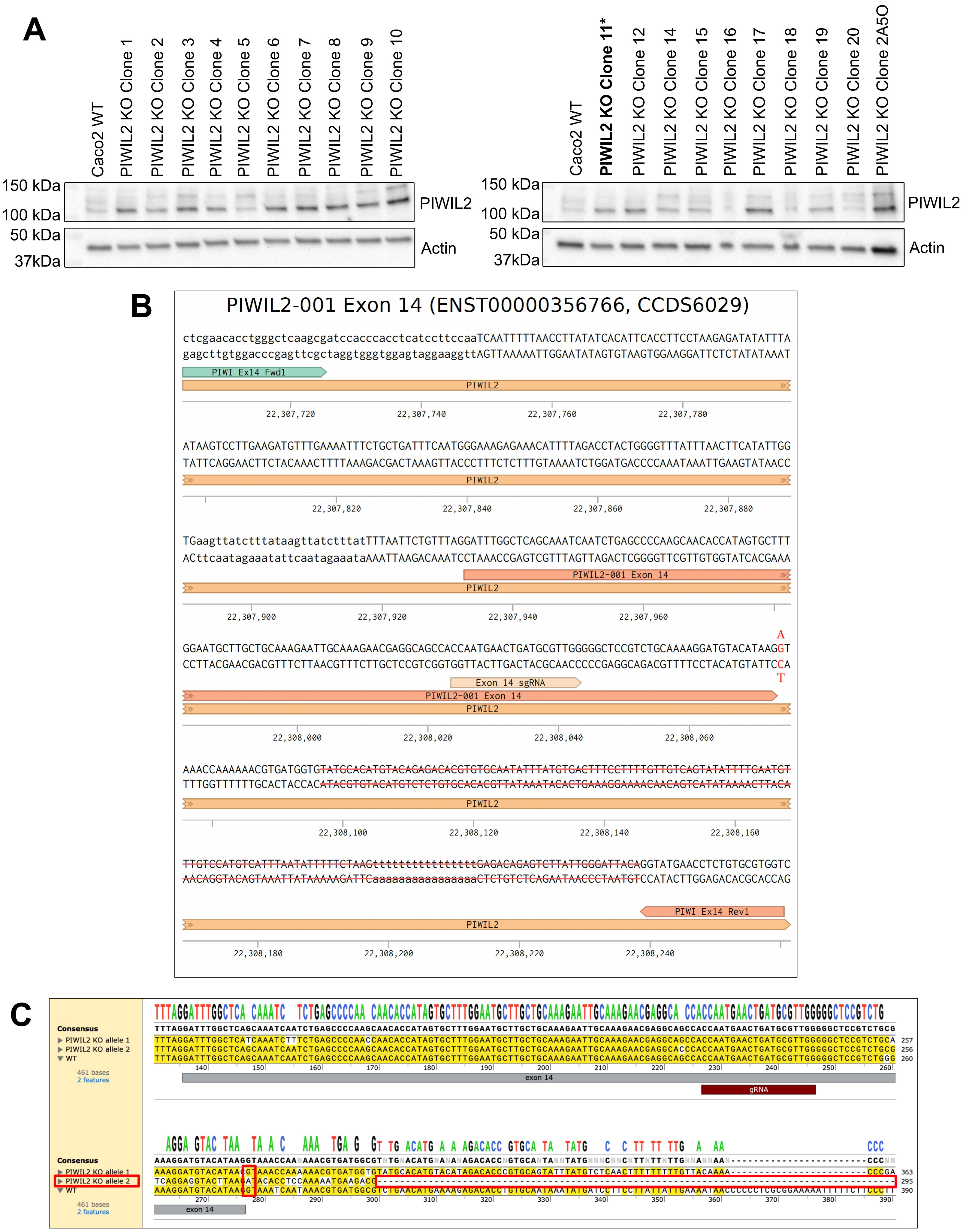

Biology Open • Supplementary information

Fig. S3. PIWIL2 KO clone selection and mutation characterization. A) Representative western blots of the screening process to identify PIWIL2 knockout clones, after transfection of Caco2 cells with CRISPR/Cas9 and a gRNA targeting PIWIL2. Clone 11 (indicated by *) was selected for subsequent studies as it was the only clone to survive selection and maintenance in cell culture. The blot lanes for Caco2 WT and Clone 11 are also shown in Fig. 5C. B) Schematic of the genomic region including exon 14 of PIWIL2 that was targeted by the gRNA, where highlighted are the genomic alterations that we identified after DNA sequencing of clone 11: a nucleotide substitution (G to A), altering the splicing target site and a large deletion within the intron. The PCR primers used to amplify and sequence the specific region are also indicated. C) Alignment of the DNA sequencing results between the WT cells and the PIWIL2 KO cells, where we identified both an unaffected allele (allele 2), as well as the actual modified allele (allele 1); modifications are indicated in red boxes.

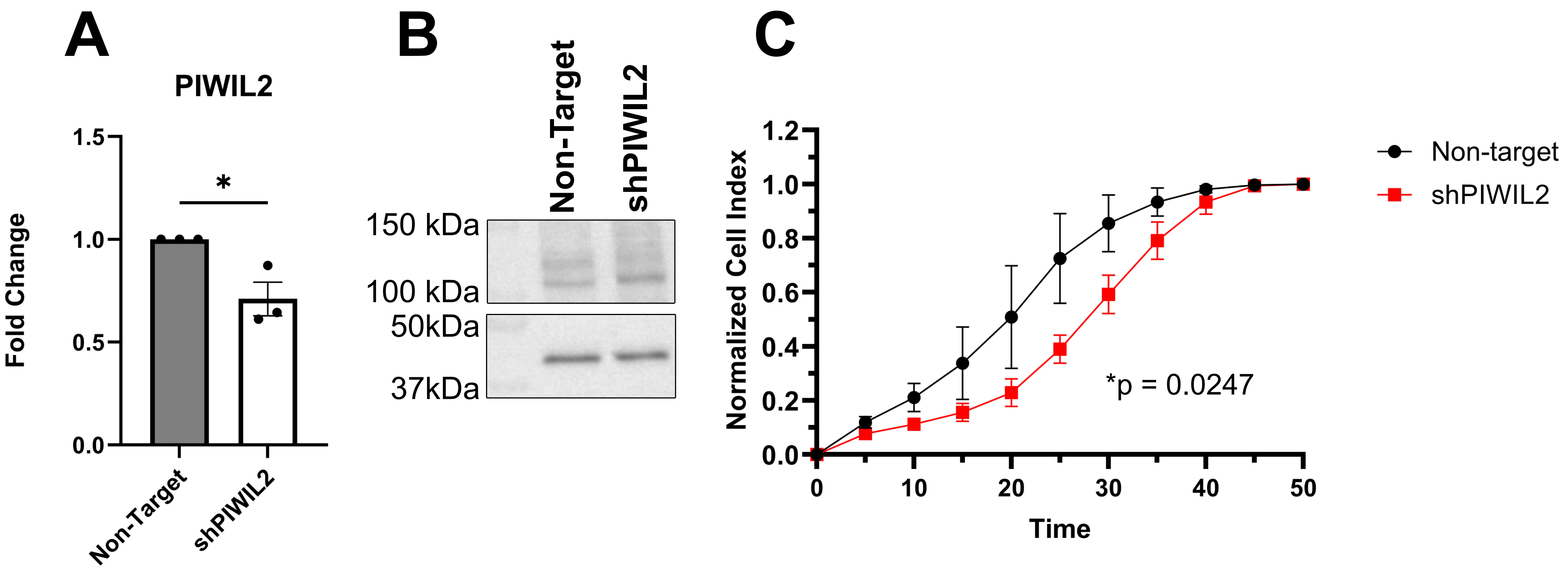

Fig. S4. Depletion of PIWIL2 by transient shRNA knockdown exhibits slowed growth in 2-dimensions (2D). A) qPCR of PIWIL2 comparing Caco2 cells treated with a Non-Target shRNA or shRNA targeting PIWIL2. Expression was normalized to 18S ribosomal RNA and fold change was calculated by ΔΔCt calculations. (n=3 biological replicates, mean ± SE, t-test *p=0.0243). B) Representative western blot of Caco2 cells treated with a Non-Target shRNA or shRNA targeting PIWIL2. C) xCELLigence cellular impedance assay to examine the proliferation rates of Caco2 cells treated with a Non-Target shRNA or shRNA targeting PIWIL2 (n=3 biological replicates, two-way repeated measures ANOVA with a Bonferroni correction for multiple comparisons, *p=0.0247).

Biology Open • Supplementary information

Table S1. PIWI mRNAs in Caco2 cells

Symbol	GeneID	avg rpms*
PIWIL1	9271	0.0
PIWIL2	55124	94.7
PIWIL3	440822	19.7
PIWIL4	143689	261.3
*avg reads/million; 3 biological replicates		

Table S2. Detailed list of the TE hits of the RNAseq sense and antisense reads presented in Fig 6 (normalized counts per million)

Available for download at
https://journals.biologists.com/bio/article-lookup/doi/10.1242/bio.061942#supplementary-data
